# Health-Related Quality of Life and Psychological Impact on Patients Post-Amputation Due to Diabetic Foot Ulcer

**DOI:** 10.7759/cureus.107290

**Published:** 2026-04-18

**Authors:** Maram Alkhatieb, Nawaf Alghamdi, Ahmad M Ghandorah, Ahmed Alshehri, Alwaleed Alamri, Majed A Almalki, Waleed M Alshehri, Khaled Alshehri

**Affiliations:** 1 General Surgery, King Abdulaziz University Faculty of Medicine, Jeddah, SAU; 2 College of Medicine, King Abdulaziz University Hospital, King Abdulaziz University Faculty of Medicine, Jeddah, SAU

**Keywords:** anxiety, depression, dfu, diabetic foot ulcer, health related-quality of life, hospital anxiety depression scale (hads), hrqol sf 36, lower limb amputation, quality of life (qol)

## Abstract

Introduction: Diabetic foot ulcers (DFUs) are a serious complication of diabetes mellitus and the leading cause of non-traumatic lower limb amputation (LLA). Such amputations can profoundly impact patients’ health-related quality of life (HRQoL) and psychological well-being. Understanding the factors that affect HRQoL and psychological well-being is crucial for developing targeted interventions to improve patient outcomes. Despite the significant burden of DFUs and their complications, which may lead to LLA, research on the HRQoL and psychological well-being of patients following LLA remains limited in the Kingdom of Saudi Arabia. This study aimed to address this gap by assessing HRQoL and psychological distress among patients who had undergone LLA due to DFU complications.

Methodology: This retrospective, cross-sectional study was conducted at King Abdulaziz University Hospital, Jeddah, Saudi Arabia. Patients who had undergone LLA due to DFU complications between 2012 and 2023, with a minimum post-amputation period of one year, were enrolled. Data collection involved a structured telephone interview, during which informed consent was obtained from all patients, and demographic and clinical information was recorded. We administered the “Short Form-36” (SF-36) and the “Hospital Anxiety and Depression Scale” (HADS) to assess the patients’ HRQoL and psychological distress status.

Results: This study included 51 patients who had undergone DFU-related LLA (median age 63.0 years; 74.5% men). The highest SF-36 scores were observed in the “social functioning,” “role limitations due to emotional problems,” and “pain” domains, while the lowest scores were in the “role limitations due to physical health,” “physical functioning,” and “energy/fatigue” domains. Depression and anxiety were common, affecting 25.5% and 23.5% of patients, respectively. Regression analyses showed that a higher income, being married, and independent mobility were associated with better HRQoL, whereas major amputation, cardiovascular disease, neuropathy, depression, and anxiety were associated with poorer outcomes.

Conclusion: Patients who had undergone LLA due to DFUs showed relatively preserved HRQoL in the “social functioning,” “role limitations due to emotional problems,” and “pain” domains, while the “role limitations due to physical health,” "physical functioning," and “energy/fatigue” domains were more impaired. Depression and anxiety were common and were associated with poorer outcomes, whereas higher income, being married, and independent mobility were associated with better HRQoL. These findings may indicate a possible role for mental health screening and supportive interventions in addressing physical and psychosocial limitations in this population. Further research is needed to develop amputation-specific instruments that better capture patient-reported outcomes and to evaluate rehabilitation and psychosocial support strategies; larger, prospective studies are needed to further evaluate these relationships and confirm these findings.

## Introduction

Diabetes mellitus affects an estimated 17.7% of the population in the Kingdom of Saudi Arabia (KSA) [1]. Among individuals with inadequate glycemic control, diabetic foot ulcers (DFUs) are one of the most frequent complications [2]. The development of DFUs is primarily driven by peripheral neuropathy and peripheral arterial disease, both of which increase the risk of infection and impaired healing and, if untreated, may progress to requiring lower limb amputation (LLA) [3]. Foot ulcers resulting from poor glycemic control are the leading cause of non-traumatic LLA [4]. The loss of a limb is a devastating event, and multiple studies have revealed that anxiety and depression are common following LLA [5]. Globally, a person loses a leg every 20 seconds, and 85% of these amputations are due to DFUs [6]. A Portuguese study of 108 patients with DFUs measured health-related quality of life (HRQoL) using the “Short Form-36” (SF-36) survey, which evaluates both physical and mental HRQoL. The study found that, at the group level, most patients experienced a significant decline in physical HRQoL after surgery, while mental HRQoL scores remained largely unchanged [7]. In KSA and the Middle East, few studies have examined HRQoL and psychological distress among patients who have undergone LLA due to DFUs. Therefore, this study aimed to examine HRQoL and psychological distress in patients who have undergone LLA due to DFU complications. We utilized the SF-36 survey to evaluate various domains of HRQoL and correlated the outcomes with patients' clinical and demographic characteristics. The assessment of anxiety and depression levels was carried out utilizing the “Hospital Anxiety and Depression Scale” (HADS).

## Materials and methods

Study design, setting, and population

This cross-sectional study was performed at King Abdulaziz University Hospital, a tertiary care center in Jeddah, KSA. We retrospectively reviewed all cases of confirmed LLA due to DFU complications between 2012 and 2023. The inclusion criteria were as follows: (1) patients who underwent LLA due to DFU complications with a minimum recovery period of one year, (2) aged 18 to 75 years, and (3) those who provided consent. The criteria for exclusion from the study were defined as follows: (1) patients with major medical conditions unrelated to diabetes mellitus, (2) amputations for medical reasons other than complicated DFU, (3) patients who underwent double limb amputation, and (4) patients who were diagnosed with psychiatric disorders and/or were taking psychiatric medication. For patients with a history of multiple LLAs due to DFU complications, we focused on their last amputation.

Data collection

Telephone calls were used to gather information related to demographic variables, including sex, date of birth, nationality, whether the patient lived in an urban or rural area, marital status, educational level, smoking status (smoker or ex-smoker), living arrangements (with family or alone), professional status, and monthly household income. In terms of clinical variables, we inquired about the diabetes type, duration since diabetes diagnosis, medication type, adherence to medication and blood sugar testing, diabetes complications (including cardiovascular diseases, nephropathy, neuropathy, and retinopathy), the ability of the patient to stand and walk alone, number of amputation surgeries, time since amputation, and level of amputation (minor: below the ankle joint; major: along the ankle joint or above). Additionally, we asked the patients whether they were aware that DFU might lead to amputation, whether they received assistance at home, whether they had been referred to social services after amputation (if yes, whether they received assistance), and whether they used a prosthetic limb. 

HRQoL and psychological distress were assessed via telephone interviews using two scales. The first scale was the SF-36 survey [8]; the Arabic version was administered to patients [9]. This survey is used to evaluate HRQoL in eight different dimensions: “physical functioning,” “role limitations due to physical health,” “bodily pain,” “general health perceptions,” “vitality,” “social functioning,” “role limitations due to emotional problems,” and “mental health.” Each domain is evaluated on a scale ranging from 0 to 100, where higher scores reflect a better perception of the health status and functional capacity within that domain.

The second scale was the Arabic version of the HADS [10,11]. Use of the HADS was authorized under license from Mapi Research Trust. The HADS consists of 14 measures: seven items are used to evaluate anxiety, and seven items are used to evaluate depression. The patients were instructed to assess the intensity of their symptoms during the previous week using a numerical scale that ranged from 0 to 3. A score of 0 signified the absence of symptoms, while a score of 3 indicated the presence of severe symptoms. The overall score for each domain (anxiety and depression) ranges from 0 to 21, where higher scores correspond to more intense symptoms.

Statistical analysis

Statistical analyses were conducted using RStudio software (R version 4.3.1). Continuous variables are summarized as either the median and interquartile range (IQR) or mean ± standard deviation, while categorical variables are expressed as the frequency and percentage. The internal consistency of the SF-36 and HADS was assessed using Cronbach’s alpha. The Mann-Whitney U test was applied for comparisons between two independent groups, while the Kruskal-Wallis test was employed for analyses involving more than two groups. Multiple linear regression analyses were performed to identify predictors of various domains of the SF-36 and HADS. Two multivariable regression models were constructed: Model 1 included all independent variables, which were incorporated using the enter method, while Model 2 included only the variables that were significantly associated with the SF-36 or HADS domain scores in the initial inferential analyses. For the purposes of this study, results yielding a p-value of less than 0.05 were interpreted as statistically significant.

Ethical approval

The Institutional Review Board of King Abdulaziz University Hospital, Jeddah, KSA, reviewed and approved the study protocol (reference number: 557-23). Verbal consent was obtained from all patients before the interviews, and they were aware of the study objectives.

## Results

Demographic characteristics

This study included 51 patients who underwent LLA due to DFUs. The median age was 63.0 years (IQR = 58.5 to 68.5 years), and 74.5% were men. Most patients were Saudi nationals (60.8%) and resided in urban areas (94.1%). A significant portion of the patients were married (78.4%), and 33.3% had a university education. Active smokers comprised 15.7%, while 47.1% were ex-smokers. A considerable majority of patients (94.1%) lived with their families. The level of employment was low; only 11.8% were employed, and 88.2% were unemployed or retired. More than half the patients had a monthly household income of ≤5000 Saudi Arabian riyals (SAR) (64.7%, Table 1).

**Table 1:** Demographic characteristics of patients The data are shown as n (%) or median (interquartile range). SAR: Saudi Arabian Riyal

Characteristic	Description
Age	63.0 (58.5–68.5)
Sex	
Male	38 (74.5%)
Female	13 (25.5%)
Nationality	
Saudi	31 (60.8%)
Non-Saudi	20 (39.2%)
Location of residence	
Urban	48 (94.1%)
Rural	3 (5.9%)
Marital status	
Single	11 (21.6%)
Married	40 (78.4%)
Educational level	
Illiterate	14 (27.5%)
Elementary	5 (9.8%)
Secondary	6 (11.8%)
High school	9 (17.6%)
University (undergraduate and postgrad)	17 (33.3%)
Active smoker	8 (15.7%)
Ex-smoker	24 (47.1%)
Live with a family	48 (94.1%)
Professional status	
Employed	6 (11.8%)
Unemployed/retired	45 (88.2%)
Monthly household income (SAR)	
≤5,000	33 (64.7%)
5,001 to 10,000	6 (11.8%)
10,001 to 20,000	9 (17.6%)
20,001 to 30,000	2 (3.9%)
>30,000	1 (2.0%)

Characteristics of patients with diabetes and LLA

Type 2 diabetes was predominant among the patients (92.2%), and 94.1% had been diagnosed for more than 10 years. The treatments varied; 47.1% of the patients used hypoglycemic agents and insulin. Most took their treatment regularly (88.2%) and tested their blood sugar at home (86.3%); 31.4% of patients measured their blood sugar level three to six times a week. Complications included cardiovascular disease (72.5%), nephropathy (29.4%), neuropathy (43.1%), and diabetes-related eye disease (68.6%). The median time since amputation surgery was 3 years (IQR = 2 to 6 years). Of the patients, 74.5% had minor amputations, and most patients had only undergone one amputation surgery (72.5%). All patients received assistance at home. However, only 17.6% were referred to social services. Of the patients, 62.7% were able to stand and walk around without assistance; 51% were aware before amputation that DFUs could lead to amputation; and prosthetic limb use was reported by 15.7% of the patients (Table 2).

**Table 2:** Characteristics of patients with diabetes who had undergone lower limb amputation Data are shown as n (%) or median (interquartile range).

Characteristic	Description
Type of diabetes	
Type 1	4 (7.8%)
Type 2	47 (92.2%)
Time since diagnosis with diabetes	
<5 years	2 (3.9%)
5 to 10 years	1 (2.0%)
>10 years	48 (94.1%)
Type of treatment for diabetes	
Hypoglycemic agents	8 (15.7%)
Insulin	19 (37.3%)
Both	24 (47.1%)
Take the treatment regularly	
No	1 (2.0%)
Sometimes	5 (9.8%)
Yes	45 (88.2%)
Test blood sugar at home	44 (86.3%)
Frequency of blood sugar testing	
No (does not test blood sugar at home)	7 (13.7%)
<3 times weekly	13 (25.5%)
3 to 6 times weekly	16 (31.4%)
Once a day	5 (9.8%)
Multiple times a day	10 (19.6%)
Diabetes-related complications	
Diabetes-related eye disease	35 (68.6%)
Cardiovascular disease	37 (72.5%)
Nephropathy	15 (29.4%)
Neuropathy	22 (43.1%)
None	6 (11.8%)
Time since the amputation surgery (years)	3.0 (2.0–6.0)
Ability to stand and walk around without assistance	32 (62.7%)
Number of amputations due to diabetic foot ulcers	
1	37 (72.5%)
2	11 (21.6%)
3	3 (5.9%)
Amputation	
Minor	38 (74.5%)
Major	13 (25.5%)
Were aware before amputation that diabetic foot ulcers may lead to amputation	26 (51.0%)
Receive assistance at home	51 (100.0%)
Referred to social services after the amputation	
No	42 (82.4%)
Yes, but I did not get assistance	4 (7.8%)
Yes, and I got assistance	5 (9.8%)
Have a prosthetic limb	8 (15.7%)

SF-36 and HADS results

HRQoL varied among the patients. The highest median values were recorded in “social functioning” (median = 100.0, IQR = 25.0 to 100.0), “role limitations due to emotional problems” (median = 100.0, IQR = 0.0 to 100.0), and “pain” (median = 87.5, IQR = 52.5 to 100.0). Conversely, the lowest median values were noted for “role limitations due to physical health” (median = 25.0, IQR = 0.0 to 100.0), “physical functioning” (median = 45.0, IQR = 15.0 to 82.5), and “energy/fatigue” (median = 50.0, IQR = 35.0 to 77.0). Cronbach’s alpha for these domains ranged from 0.688 for “pain” to 0.999 for “role limitations due to emotional problems,” indicating acceptable to excellent reliability across all domains (Table 3).

**Table 3:** SF-36 and HADS results * Indicates subdomains of the HADS scale; otherwise, subdomains of the SF-36 questionnaire are included. HADS: Hospital Anxiety and Depression Scale; IQR: interquartile range; SD: standard deviation; SF-36: Short Form-36.

Characteristic	Median (IQR)	Mean ± SD	Min–Max	Cronbach’s Alpha	No. of Items
Physical functioning	45.0 (15.0–82.5)	45.9 ± 36.6	0.0–100.0	0.941	10
Role limitations due to physical health	25.0 (0.0–100.0)	46.1 ± 47.8	0.0–100.0	0.966	4
Role limitations due to emotional problems	100.0 (0.0–100.0)	60.8 ± 49.3	0.0–100.0	0.999	3
Energy/fatigue	50.0 (35.0–70.0)	53.1 ± 22.9	5.0–100.0	0.788	4
Emotional well-being	68.0 (48.0–84.0)	65.8 ± 23.5	0.0–100.0	0.857	5
Social functioning	100.0 (25.0–100.0)	67.6 ± 39.0	0.0–100.0	0.967	2
Pain	87.5 (52.5–100.0)	73.8 ± 28.9	0.0–100.0	0.688	2
General health	60.0 (40.0–77.5)	59.3 ± 24.0	20.0–100.0	0.842	5
Depression^*^	3.0 (0.0–11.0)	5.8 ± 6.3	0.0–20.0	0.937	7
Anxiety^*^	4.0 (0.5–7.5)	5.3 ± 5.5	0.0–18.0	0.916	7

In terms of the HADS results, the median score for depression was 3.0 (IQR = 0.0 to 11.0), and the mean was 5.8 ± 6.3. The median score for anxiety was 4.0 (IQR = 0.5 to 7.5), and the mean was 5.3 ± 5.5. Cronbach’s alpha coefficients for the depression and anxiety subscales were 0.937 and 0.916, respectively, reflecting high internal consistency reliability (Table 3).

**Figure 1:**
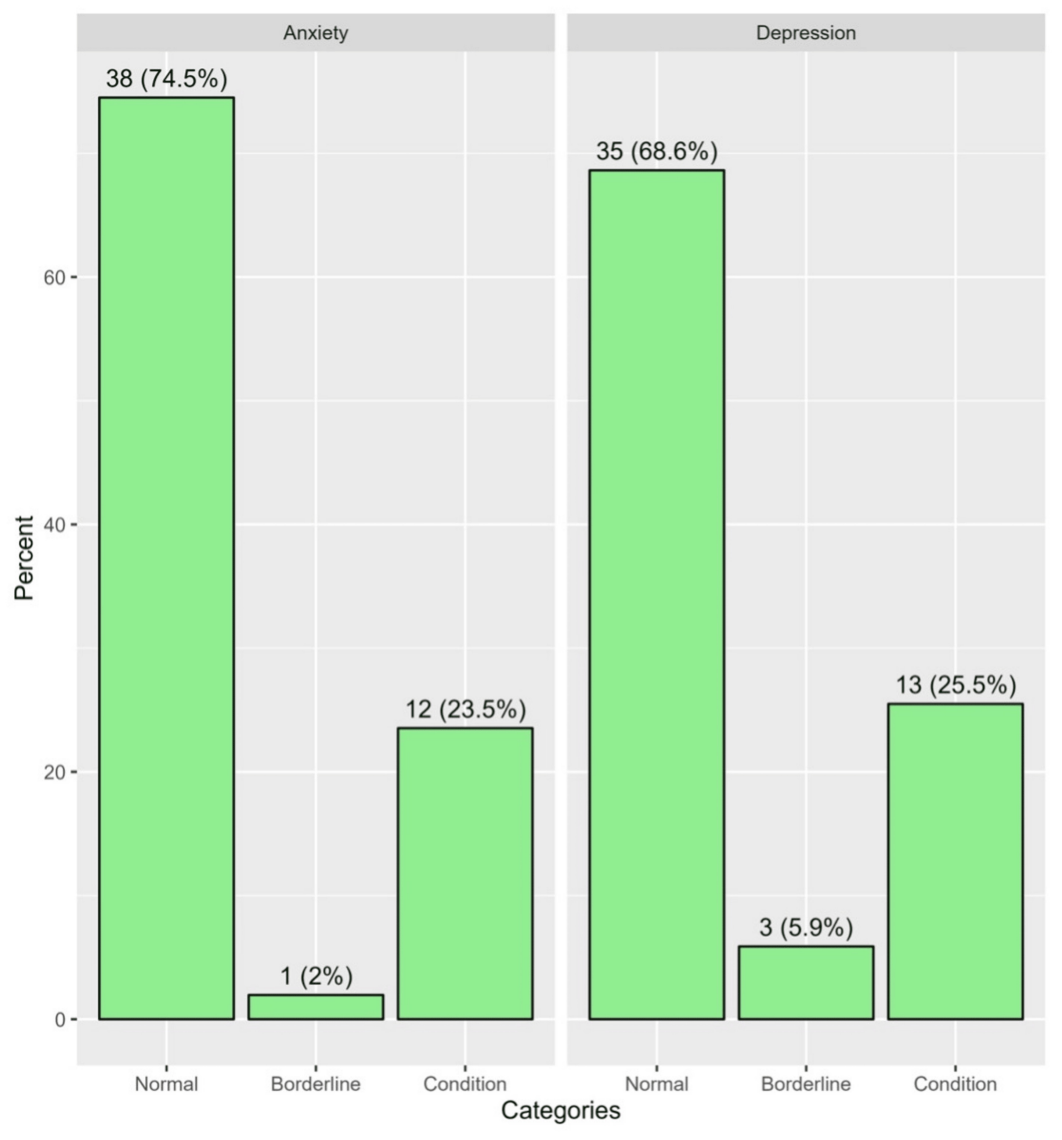
Distribution of the depression and anxiety categories based on the Hospital Anxiety and Depression Scale scores and categorization criteria

Figure 1 illustrates the distribution of depression and anxiety categories among patients according to the HADS scoring criteria. For depression, 5.9% of patients were classified as borderline, and 25.5% were identified as having a significant depressive condition. Regarding anxiety, 74.5% of patients were in the normal category, 2.0% of patients were in the borderline category, and 23.5% were identified as having significant anxiety.

Predictors of HRQoL

The multivariable regression analysis of “physical functioning” revealed that a monthly household income of 5001 to 10000 SAR was a significant predictor of higher “physical functioning” compared to the reference category of ≤5000 SAR in Model 1 (beta = 38.2, 95% confidence interval [CI] = 4.16 to 72.3, p = 0.038) (Table 4). The significance was maintained in Model 2, with a slightly lower beta value (beta = 23.0, 95% CI = 1.76 to 44.3, p = 0.041). The ability to stand and walk without assistance was another significant predictor in Model 1 (beta = 43.7, 95% CI = 25.0 to 62.4, p < 0.001) and remained significant in Model 2 (beta = 37.4, 95% CI = 22.4 to 52.4, p < 0.001). Additionally, major amputation was significant in Model 1, predicting lower “physical functioning” scores compared to minor amputations (beta = -35.6, 95% CI = -68.4 to -2.76, p = 0.044); however, this association was not significant in Model 2 (beta = -13.8, 95% CI = -29.3 to 1.63, p = 0.088).

**Table 4:** Predictors of physical functioning Model 1 included all independent variables using the enter method, whereas Model 2 included the variables that were significant in the univariable analysis. CI: confidence interval; SAR: Saudi Arabian Riyal. *p<0.05, **p≤0.001

	Model 1			Model 2		
Characteristic	Beta	95%CI	p-value	Beta	95%CI	p-value
Age (year increment)	-0.28	-2.09, 1.53	0.764			
Sex (female)	-10.5	-41.1, 20.0	0.506			
Nationality (non-Saudi)	14.1	-5.26, 33.4	0.167			
Marital status (married)	10.7	-12.6, 34.0	0.377	13.1	-2.54, 28.8	0.109
Educational level						
Illiterate	Reference	Reference				
Elementary	6.29	-25.6, 38.2	0.703			
Secondary	-21.1	-56.7, 14.4	0.255			
High school	-19.9	-52.1, 12.2	0.236			
University (undergraduate and postgrad)	-17.3	-49.9, 15.2	0.307			
Professional status (unemployed/retired)	2.57	-35.7, 40.8	0.897			
Monthly household income (SAR)						
≤5,000	Reference	Reference		Reference	Reference	
5,001 to 10,000	38.2	4.16, 72.3	0.038*	23.0	1.76, 44.3	0.041*
10,001 to 20,000	23.0	-3.97, 50.0	0.108	10.6	-6.87, 28.2	0.241
20,001 to 30,000	30.9	-16.7, 78.6	0.215	18.0	-14.7, 50.8	0.288
>30,000	-18.3	-87.7, 51.1	0.610	-33.5	-78.5, 11.4	0.152
Diabetes-related complications						
Cardiovascular disease	-3.33	-23.3, 16.7	0.747	-9.51	-24.6, 5.56	0.224
Nephropathy	12.3	-9.45, 34.1	0.278			
Neuropathy	-8.38	-30.2, 13.4	0.458	-11.9	-28.5, 4.70	0.169
Diabetes-related eye disease	-5.41	-25.3, 14.5	0.599	-4.89	-23.0, 13.2	0.599
Ability to stand and walk around without assistance	43.7	25.0, 62.4	<0.001**	37.4	22.4, 52.4	<0.001**
Number of amputations due to diabetic foot ulcers (continuous)	-4.53	-20.2, 11.2	0.577			
Time (in years) since the amputation surgery	3.90	0.04, 7.76	0.059			
Major amputation	-35.6	-68.4, -2.76	0.044*	-13.8	-29.3, 1.63	0.088
Have a prosthetic limb	21.6	-16.4, 59.7	0.276			
Anxiety						
Normal	Reference	Reference		Reference	Reference	
Borderline	-41.7	-101, 17.2	0.178	-18.2	-68.5, 32.2	0.484
Condition	-14.8	-42.2, 12.6	0.300	-12.3	-32.5, 7.95	0.242
Depression						
Normal	Reference	Reference		Reference	Reference	
Borderline	16.4	-16.4, 49.2	0.337	13.0	-17.6, 43.5	0.412
Condition	19.5	-7.78, 46.7	0.174	3.37	-16.7, 23.5	0.744

Regarding “physical role functioning” (Table 5), no significant predictors were identified in Model 1. However, the presence of cardiovascular disease was a significant predictor of lower “physical role functioning” compared to those without cardiovascular disease in Model 2 (beta = -35.7, 95% CI = -59.2 to -12.1, p = 0.005). Conversely, a monthly household income of 5001 to 10000 SAR was a significant predictor of higher “physical role functioning” compared to the reference category of ≤5000 SAR in Model 2 (beta = 40.9, 95% CI = 9.82 to 71.9, p = 0.014). Additionally, depression was a significant predictor of lower “physical role functioning” compared to the reference category of normal depression status in Model 2 (beta = -28.8, 95% CI = -52.2 to -5.46, p = 0.020), while it was not significant in Model 1 (beta = -10.0, 95% CI = -57.4 to 37.4, p = 0.683).

**Table 5:** Predictors of physical role functioning Model 1 included all independent variables using the enter method, whereas Model 2 included the variables that were significant in the univariable analysis. CI: confidence interval; SAR: Saudi Arabian Riyal. *p<0.05, **p≤0.01

	Model 1			Model 2		
Characteristic	Beta	95%CI	p-value	Beta	95%CI	p-value
Age (year increment)	-0.41	-3.55, 2.74	0.802			
Sex (female)	-11.0	-64.2, 42.3	0.690			
Nationality (non-Saudi)	11.9	-21.7, 45.5	0.494			
Marital status (married)	-3.91	-44.5, 36.7	0.852			
Educational level						
Illiterate	Reference	Reference				
Elementary	9.09	-46.4, 64.6	0.751			
Secondary	-8.91	-70.8, 53.0	0.780			
High school	-23.2	-79.2, 32.8	0.425			
University (undergraduate and postgrad)	-18.2	-74.9, 38.5	0.535			
Professional status (unemployed/retired)	-35.9	-103, 30.6	0.301			
Monthly household income (SAR)						
≤5,000	Reference	Reference		Reference	Reference	
5,001 to 10,000	39.1	-20.2, 98.4	0.209	40.9	9.82, 71.9	0.014*
10,001 to 20,000	4.93	-42.1, 51.9	0.839	-3.74	-28.4, 20.9	0.768
20,001 to 30,000	31.5	-51.4, 114	0.463	38.5	-10.2, 87.1	0.129
>30,000	-57.3	-178, 63.4	0.361	-22.9	-90.6, 44.7	0.511
Diabetes-related complications						
Cardiovascular disease	-33.5	-68.3, 1.34	0.072	-35.7	-59.2, -12.1	0.005**
Nephropathy	-10.2	-48.1, 27.8	0.604	-11.1	-34.6, 12.4	0.361
Neuropathy	18.9	-19.0, 56.8	0.338	3.06	-21.4, 27.6	0.808
Diabetes-related eye disease	-26.5	-61.1, 8.13	0.147	-20.1	-45.7, 5.59	0.133
Ability to stand and walk around without assistance	25.1	-7.46, 57.7	0.144	17.2	-4.72, 39.0	0.132
Number of amputations due to diabetic foot ulcers (continuous)	-1.09	-28.4, 26.2	0.938			
Time (in years) since the amputation surgery	4.29	-2.43, 11.0	0.223			
Major amputation	-13.9	-71.0, 43.3	0.638			
Have a prosthetic limb	-0.02	-66.3, 66.2	>0.999			
Anxiety						
Normal	Reference	Reference				
Borderline	-4.91	-108, 97.7	0.926			
Condition	-12.8	-60.4, 34.8	0.603			
Depression						
Normal	Reference	Reference		Reference	Reference	
Borderline	-23.7	-80.7, 33.4	0.424	-29.1	-69.8, 11.6	0.169
Condition	-10.0	-57.4, 37.4	0.683	-28.8	-52.2, -5.46	0.020*

Concerning “emotional role functioning” (Table 6), marriage was a significant predictor of higher “emotional role functioning” compared to being single in Model 2 (beta = 29.1, 95% CI = 4.57 to 53.5, p = 0.026). However, it was not significant in Model 1 (beta = 33.0, 95% CI = -9.43 to 75.5, p = 0.140). Borderline depression was a significant predictor of lower “emotional role functioning” compared to a normal depression status in Model 2 (beta = -56.9, 95% CI = -104 to -9.87, p = 0.023), while it was not significant in Model 1 (beta = -47.0, 95% CI = -107 to 12.7, p = 0.136). Additionally, depression significantly predicted lower “emotional role functioning” in Model 2 (beta = -46.8, 95% CI = -78.5 to -15.1, p = 0.006).

**Table 6:** Predictors of emotional role functioning Model 1 involved all independent variables using the enter method, whereas Model 2 included the variables that were significant in the univariable analysis. CI: confidence interval; SAR: Saudi Arabian Riyal. *p<0.05, **p≤0.01

	Model 1			Model 2		
Characteristic	Beta	95%CI	p-value	Beta	95%CI	p-value
Age (year increment)	-1.23	-4.52, 2.06	0.471			
Sex (female)	-11.9	-67.6, 43.7	0.678			
Nationality (non-Saudi)	2.71	-32.5, 37.9	0.881			
Marital status (married)	33.0	-9.43, 75.5	0.140	29.1	4.57, 53.5	0.026*
Educational level						
Illiterate	Reference	Reference				
Elementary	-18.5	-76.7, 39.6	0.538			
Secondary	-32.3	-97.1, 32.5	0.338			
High school	-31.1	-89.7, 27.5	0.308			
University (undergraduate and postgrad)	-9.97	-69.3, 49.3	0.745			
Professional status (unemployed/retired)	-12.3	-82.0, 57.4	0.733	-14.7	-56.2, 26.8	0.492
Monthly household income (SAR)						
≤5,000	Reference	Reference		Reference	Reference	
5,001 to 10,000	16.7	-45.4, 78.7	0.603	16.3	-22.8, 55.5	0.419
10,001 to 20,000	9.86	-39.3, 59.0	0.698	16.8	-11.3, 44.9	0.250
20,001 to 30,000	16.5	-70.2, 103	0.712	25.9	-30.8, 82.6	0.376
>30,000	-22.6	-149, 104	0.729	-13.7	-98.5, 71.0	0.753
Diabetes-related complications						
Cardiovascular disease	-14.0	-50.4, 22.5	0.460	-17.6	-41.9, 6.68	0.164
Nephropathy	-6.55	-46.3, 33.2	0.749			
Neuropathy	13.2	-26.5, 52.8	0.521			
Diabetes-related eye disease	-18.2	-54.5, 18.0	0.334	-8.97	-32.0, 14.0	0.449
Ability to stand and walk around without assistance	15.9	-18.2, 50.0	0.370	16.4	-7.28, 40.0	0.183
Number of amputations due to diabetic foot ulcers (continuous)	-3.33	-31.9, 25.2	0.821			
Time (in years) since the amputation surgery	0.38	-6.65, 7.41	0.917			
Major amputation	2.24	-57.6, 62.0	0.942			
Have a prosthetic limb	7.21	-62.1, 76.5	0.840			
Anxiety						
Normal	Reference	Reference		Reference	Reference	
Borderline	-30.2	-138, 77.2	0.587	-42.4	-121, 36.3	0.298
Condition	4.91	-44.9, 54.8	0.849	7.59	-23.5, 38.7	0.635
Depression						
Normal	Reference	Reference		Reference	Reference	
Borderline	-47.0	-107, 12.7	0.136	-56.9	-104, -9.87	0.023*
Condition	-39.9	-89.6, 9.75	0.128	-46.8	-78.5, -15.1	0.006**

As shown in Table 7, anxiety was a significant predictor of lower “emotional well-being” compared to a normal anxiety status in Model 1 (beta = -21.7, 95% CI = -39.0 to -4.41, p = 0.021) and Model 2 (beta = -24.6, 95% CI = -40.0 to -9.08, p = 0.004). Borderline depression was also a significant predictor of lower “emotional well-being” compared to a normal depression status in both Model 1 (beta = -32.3, 95% CI = -53.0 to -11.6, p = 0.005) and Model 2 (beta = -32.6, 95% CI = -51.8 to -13.4, p = 0.002).

**Table 7:** Predictors of emotional well-being Model 1 involved all independent variables using the enter method, whereas Model 2 included all variables that were significant in the univariable analysis. CI: confidence interval; SAR: Saudi Arabian Riyal. *p<0.05, **p≤0.01

	Model 1			Model 2		
Characteristic	Beta	95%CI	p-value	Beta	95%CI	p-value
Age (year increment)	-0.21	-1.35, 0.93	0.725			
Sex (female)	8.02	-11.3, 27.3	0.424	6.08	-6.18, 18.3	0.338
Nationality (non-Saudi)	-7.12	-19.3, 5.08	0.264	-6.71	-15.7, 2.33	0.155
Marital status (married)	11.5	-3.26, 26.2	0.140	6.64	-4.92, 18.2	0.268
Educational level						
Illiterate	Reference	Reference				
Elementary	-0.08	-20.2, 20.1	0.994			
Secondary	-12.6	-35.1, 9.84	0.282			
High school	-3.19	-23.5, 17.1	0.761			
University (undergraduate and postgrad)	-0.67	-21.2, 19.9	0.950			
Professional status (unemployed/retired)	-8.93	-33.1, 15.2	0.476	-12.8	-29.9, 4.21	0.149
Monthly household income (SAR)						
≤5,000	Reference	Reference		Reference	Reference	
5,001 to 10,000	-5.05	-26.6, 16.5	0.649	-5.33	-22.3, 11.7	0.543
10,001 to 20,000	6.27	-10.8, 23.3	0.478	7.85	-4.68, 20.4	0.228
20,001 to 30,000	1.00	-29.1, 31.1	0.949	3.68	-19.9, 27.3	0.761
>30,000	2.69	-41.1, 46.5	0.905	-4.41	-38.3, 29.5	0.800
Diabetes-related complications						
Cardiovascular disease	-8.29	-20.9, 4.35	0.211	-8.68	-18.8, 1.45	0.102
Nephropathy	-4.72	-18.5, 9.05	0.508	-5.47	-16.9, 5.91	0.353
Neuropathy	-9.90	-23.6, 3.84	0.171	-5.20	-15.2, 4.84	0.317
Diabetes-related eye disease	8.00	-4.55, 20.6	0.224			
Ability to stand and walk around without assistance	0.24	-11.6, 12.1	0.968	1.58	-8.33, 11.5	0.757
Number of amputations due to diabetic foot ulcers (continuous)	2.53	-7.37, 12.4	0.622	2.84	-4.98, 10.7	0.481
Time (in years) since the amputation surgery	-0.32	-2.75, 2.12	0.801			
Major amputation	5.61	-15.1, 26.3	0.601			
Have a prosthetic limb	3.16	-20.9, 27.2	0.799			
Anxiety						
Normal	Reference	Reference		Reference	Reference	
Borderline	-14.8	-52.1, 22.4	0.443	-14.6	-47.7, 18.5	0.393
Condition	-21.7	-39.0, -4.41	0.021*	-24.6	-40.0, -9.08	0.004**
Depression						
Normal	Reference	Reference		Reference	Reference	
Borderline	-32.3	-53.0, -11.6	0.005**	-32.6	-51.8, -13.4	0.002**
Condition	-6.99	-24.2, 10.2	0.434	-3.84	-17.5, 9.83	0.586

Finally, regarding “general health perceptions” (Table 8), female sex was a significant predictor of better “general health perceptions” compared to male sex in Model 1 (beta = 24.9, 95% CI = 2.67 to 47.2, p = 0.038). Neuropathy was a significant predictor of lower “general health perceptions” compared to those without this complication in Model 1 (beta = -25.0, 95% CI = -40.8 to -9.11, p = 0.005) and Model 2 (beta = -15.3, 95% CI = -29.5 to -1.11, p=0.041). Anxiety was another significant predictor of lower “general health perceptions” compared to a normal anxiety status in Model 1 (beta = -27.0, 95% CI = -46.9 to -7.05, p = 0.014); however, this was not significant in Model 2 (beta = -16.1, 95% CI = -32.4 to 0.14, p = 0.059).

**Table 8:** Predictors of the general health perceptions Model 1 involved all independent variables using the enter method, whereas Model 2 included the variables that were significant in the univariable analysis. CI: confidence interval; SAR: Saudi Arabian Riyal. *p<0.05, **p≤0.01

	Model 1			Model 2		
Characteristic	Beta	95%CI	p-value	Beta	95%CI	p-value
Age (year increment)	-0.29	-1.60, 1.03	0.671			
Sex (female)	24.9	2.67, 47.2	0.038*			
Nationality (non-Saudi)	-13.1	-27.2, 0.99	0.081			
Marital status (married)	17.6	0.65, 34.6	0.053	5.44	-6.97, 17.8	0.396
Educational level						
Illiterate	Reference	Reference				
Elementary	15.5	-7.76, 38.7	0.204			
Secondary	13.2	-12.7, 39.1	0.327			
High school	13.1	-10.3, 36.5	0.284			
University (undergraduate and postgrad)	10.2	-13.5, 33.9	0.407			
Professional status (unemployed/retired)	15.3	-12.6, 43.2	0.293			
Monthly household income (SAR)						
≤5,000	Reference	Reference				
5,001 to 10,000	-18.4	-43.2, 6.39	0.159			
10,001 to 20,000	-1.01	-20.7, 18.7	0.921			
20,001 to 30,000	21.6	-13.1, 56.3	0.234			
>30,000	28.7	-21.8, 79.2	0.277			
Diabetes-related complications						
Cardiovascular disease	-2.02	-16.6, 12.6	0.789	-3.37	-15.2, 8.49	0.581
Nephropathy	-7.52	-23.4, 8.36	0.363	-10.1	-23.7, 3.56	0.155
Neuropathy	-25.0	-40.8, -9.11	0.005**	-15.3	-29.5, -1.11	0.041
Diabetes-related eye disease	10.8	-3.71, 25.3	0.158	2.80	-10.7, 16.3	0.685
Ability to stand and walk around without assistance	9.13	-4.50, 22.8	0.202	6.59	-4.57, 17.7	0.254
Number of amputations due to diabetic foot ulcers (continuous)	-9.78	-21.2, 1.64	0.106			
Time (in years) since the amputation surgery	-2.55	-5.36, 0.26	0.089			
Major amputation	14.8	-9.10, 38.7	0.236			
Have a prosthetic limb	-8.57	-36.3, 19.2	0.550			
Anxiety						
Normal	Reference	Reference		Reference	Reference	
Borderline	-22.9	-65.8, 20.1	0.308	-7.74	-45.7, 30.3	0.692
Condition	-27.0	-46.9, -7.05	0.014*	-16.1	-32.4, 0.14	0.059
Depression						
Normal	Reference	Reference		Reference	Reference	
Borderline	-9.59	-33.5, 14.3	0.439	-18.6	-41.3, 4.12	0.117
Condition	-8.25	-28.1, 11.6	0.423	-6.28	-21.5, 8.94	0.423

## Discussion

This study aimed to evaluate the HRQoL and psychological distress of patients who had undergone LLA due to DFU complications. 

Across the eight SF-36 domains, “role limitations due to physical health,” “physical functioning,” and “energy/fatigue” were the most adversely affected, reflecting a substantial negative impact on overall HRQoL. These findings are consistent with a systematic review of patient-reported outcomes in lower limb amputees, which emphasized mobility as a central determinant of post-amputation quality of life deterioration [12]. Together, these findings highlight the importance of addressing physical health limitations and prioritizing strategies to optimize post-amputation outcomes.

In the present study, major amputations were associated with significantly reduced “physical functioning” scores compared with minor amputations, with “physical functioning” being a key domain of HRQoL. This finding aligns with a previous study of 1,088 patients, which reported that major amputation predicted lower HRQoL [13]. These results highlight the need for targeted rehabilitation and mobility-focused interventions to preserve and improve mobility and daily functional ability, particularly in patients undergoing major amputations.

Patients who could stand and walk without assistance demonstrated better “physical functioning” than those who could not. A retrospective case-control study compared patients who had undergone amputation due to DFU with patients with chronic foot ulcers who had not undergone amputation and found that mobile amputees had better HRQoL than patients with active foot ulcers, though their HRQoL remained lower than that of diabetic patients without a history of foot ulceration [14]. These findings underscore the varying degrees of physical and functional outcomes depending on the presence and severity of foot complications and the need for amputation in diabetic patients.

Diabetes-related complications, including cardiovascular disease and neuropathy, were negatively associated with HRQoL domains, specifically “physical role functioning” and “general health perceptions.” The significant correlation between cardiovascular disease and diminished “physical role functioning” underscores the importance of holistic patient care, suggesting that managing cardiovascular health is essential for enhancing “physical role functioning” and overall HRQoL. Neuropathy was also significantly associated with lower “general health perceptions,” consistent with a cross-sectional study of 304 high-risk diabetes patients, which found “general health perceptions” among the lowest-scoring SF-36 domains in patients with peripheral neuropathy [15].

Our findings suggest that patients with a higher income tend to have better “physical functioning” and “physical role functioning,” likely because greater financial resources enable access to rehabilitation, assistive devices, and follow-up care. This aligns with studies showing that financial hardship in amputees is linked to poorer “physical functioning,” underscoring how socioeconomic advantage can directly impact post-amputation mobility [16].

In this study, HADS results revealed a mean depression score of 5.8 ± 6.3 and a mean anxiety score of 5.3 ± 5.5. These scores were lower than those reported in a 2019 longitudinal study assessing the psychological impact of amputation in 144 patients with diabetes, which found mean anxiety and depression scores of 7.74 ± 4.91 and 7.85 ± 6.13, respectively, ten months post-amputation [17]. This suggests that the timing of assessment may influence reported psychological outcomes, as our population was assessed at least one year post-amputation. Further, compared to a 2021 cross-sectional study of 250 patients with DFU (62 with LLA), our patients also had lower anxiety and depression scores (2021 study results: mean anxiety 8.52 ± 4.1; mean depression 9.15 ± 4.3) [18], indicating less pronounced psychological distress in our cohort. However, direct comparison is limited because most patients in that study did not undergo amputation.

Depression was linked to poorer “physical functioning” and “emotional role functioning,” while borderline depression correlated with lower “emotional role functioning” and “emotional well-being.” Anxiety was linked to worse “emotional well-being” and “general health perceptions.” The impact of depression on “physical role functioning” may reflect psychomotor effects [19], where fatigue, lack of energy, and reduced motivation negatively affect HRQoL. These findings are in agreement with previous studies showing that depression, with or without anxiety, adversely affects HRQoL [20]. Additionally, patients who had undergone transtibial amputation were found to have worse “emotional role functioning” and “physical functioning” compared to patients with chronic foot ulcers [14]. These findings highlight the importance of screening for anxiety and depression before and after amputation to enable timely psychological evaluation and treatment in order to enhance both psychological well-being and overall HRQoL.

“Emotional role functioning” was also associated with marital status, with married individuals scoring higher than single individuals, suggesting that spousal support alleviates psychological stress. This finding aligns with a cross-sectional study of 525 patients with DFUs, which reported lower HRQoL scores in patients living alone compared to those living with a partner [21].

Finally, only 51% of patients were aware that DFUs could lead to amputation, highlighting the importance of patient education. Awareness of DFU complications is associated with prompt medical consultation, adherence to care, and better treatment compliance. For example, a recent randomized controlled trial demonstrated that structured health education significantly improved patients’ awareness and foot self-care practices, with higher awareness correlating with better self-care behaviors, underscoring the critical role of patient education in preventing complications [22].

Strengths and limitations

A major strength of this study is the use of validated and reliable instruments (SF-36 and HADS), which allowed for robust assessment of the physical and mental health domains. Another strength is that it addresses a critical gap in the literature, as research on HRQoL after LLA due to DFUs in Saudi Arabia is very limited. However, because these tools are generic, they may not fully capture amputation, specific challenges such as phantom limb pain, and prosthesis adaptation, which is an important limitation noted for future research. Additionally, the use of telephone interviews with recall of clinical variables may introduce recall bias. Other limitations include the modest sample size, single-center design, and limited representation of prosthesis users, which may restrict generalizability and causal interpretation. The study may also be subject to selection and survivorship bias, as only patients who were reachable, consented, and able to participate in telephone interviews were included. Furthermore, the exclusion of patients with diagnosed psychiatric disorders or those receiving psychiatric treatment may limit generalizability and affect the estimation of psychological distress in this population. Finally, given the modest sample size relative to the number of variables analyzed, the regression findings should be interpreted as exploratory and may be subject to model instability.

## Conclusions

In this study, patients scored highest in the “social functioning,” “role limitations due to emotional problems,” and “pain” domains, indicating relatively preserved HRQoL in these areas, while the “role limitations due to physical health,” “physical functioning,” and “energy/fatigue” domains appeared more affected. Depression and anxiety were relatively common and were associated with poorer outcomes, whereas higher income, being married, and independent mobility were associated with better HRQoL. These findings suggest potential associations between clinical, functional, and psychosocial factors and HRQoL in patients with DFU undergoing LLA; however, they should be interpreted with appropriate caution given the exploratory nature of the study, modest sample size, and analytical scope. These results may indicate a potential role for mental health screening and supportive interventions in this population. Future studies with larger, prospective designs are needed to further evaluate these relationships and confirm these findings. 
